# 4.C. Workshop: Promoting health without borders: cross-border public health policy in the Euregio Meuse-Rhine

**DOI:** 10.1093/eurpub/ckac129.213

**Published:** 2022-10-25

**Authors:** Organised by: EUPHA-PHPP, Gesundheitsamt Düren (Germany), Foundation euPrevent (Netherlands), Chair persons: Sofia Ribeiro (EUPHA-PHPP)

## Abstract

For more than 20 years, different public health institutions in the German-Belgian-Dutch border triangle have been involved in cross-border public health activities. The partners in the Euregio Meuse-Rhine (EMR), as this area is called, have jointly implemented studies on COVID-19 and on adolescent risk behaviour. They have established joint health reporting for various purposes and implemented prevention measures together. The special problems in the border regions during the Corona pandemic have once again impressively shown how important cross-border cooperation is - also for cross-border public health policy. In the health sector, orientation towards municipal, state and federal borders does not lead to the desired results. The results of the Euregional COVID 19 study of 2021 show that in this way a ‘borderless’ life - and partly also cross-border health care - cannot be adequately served. In fact, in the everyday life of a border community, there is hardly any difference between a district border and a national border. In the EMR, this is exemplary for the entire European Union, with its many national or local responsibilities. For infectious diseases, lifestyle risks, environmental toxins or climate risks, borders have no meaning. For health, however, they do. Cross-border policy and politics is the appropriate response to real European conditions. The workshop will show the possibilities and results of cross-border policy on the basis of 3 examples from the long-standing cooperation of public health actors from the Euregio Meuse-Rhine. Finally, we will present these factors and put them up for discussion. From these and other activities, the factors that enable or hinder policy along borders can be deduced. We will present these factors, classify their significance and present the possibility of generalisation for cross-border work for discussion.

**Key messages:**

• Cross-border policy and coordination are the appropriate responses to the current realities in the European Union.

• National differences in culture, administration and policy can be obstacles to cooperation; but are usually inspiration for new approaches and input for best practice.

